# An UPLC-MS/MS method for highly sensitive high-throughput analysis of phytohormones in plant tissues

**DOI:** 10.1186/1746-4811-8-47

**Published:** 2012-11-22

**Authors:** Gerd Ulrich Balcke, Vinzenz Handrick, Nick Bergau, Mandy Fichtner, Anja Henning, Hagen Stellmach, Alain Tissier, Bettina Hause, Andrej Frolov

**Affiliations:** 1 Department of Cell and Metabolic Biology, Leibniz Institute of Plant Biochemistry, Weinberg 3, Halle (Saale), 06120, Germany; 2 Present address: Max Planck Institute for Chemical Ecology, Department of Biochemistry, Hans-Knoell-Str. 8, Jena, 07745, Germany; 3 Faculty of Chemistry and Mineralogy, Institute of Bioanalytical Chemistry, Centre for Biotechnology and Biomedicine, Leipzig University, Deutscher Platz 5, Leipzig, 04103, Germany

**Keywords:** Phytohormones, Jasmonates, LC-MS/MS, Solid phase extraction (SPE), Quantification, Electrospray ionization, Plant stress response, Wounding

## Abstract

**Background:**

Phytohormones are the key metabolites participating in the regulation of multiple functions of plant organism. Among them, jasmonates, as well as abscisic and salicylic acids are responsible for triggering and modulating plant reactions targeted against pathogens and herbivores, as well as resistance to abiotic stress (drought, UV-irradiation and mechanical wounding). These factors induce dramatic changes in phytohormone biosynthesis and transport leading to rapid local and systemic stress responses. Understanding of underlying mechanisms is of principle interest for scientists working in various areas of plant biology. However, highly sensitive, precise and high-throughput methods for quantification of these phytohormones in small samples of plant tissues are still missing.

**Results:**

Here we present an LC-MS/MS method for fast and highly sensitive determination of jasmonates, abscisic and salicylic acids. A single-step sample preparation procedure based on mixed-mode solid phase extraction was efficiently combined with essential improvements in mobile phase composition yielding higher efficiency of chromatographic separation and MS-sensitivity. This strategy resulted in dramatic increase in overall sensitivity, allowing successful determination of phytohormones in small (less than 50 mg of fresh weight) tissue samples. The method was completely validated in terms of analyte recovery, sensitivity, linearity and precision. Additionally, it was cross-validated with a well-established GC-MS-based procedure and its applicability to a variety of plant species and organs was verified.

**Conclusion:**

The method can be applied for the analyses of target phytohormones in small tissue samples obtained from any plant species and/or plant part relying on any commercially available (even less sensitive) tandem mass spectrometry instrumentation.

## Background

**Figure 1:**
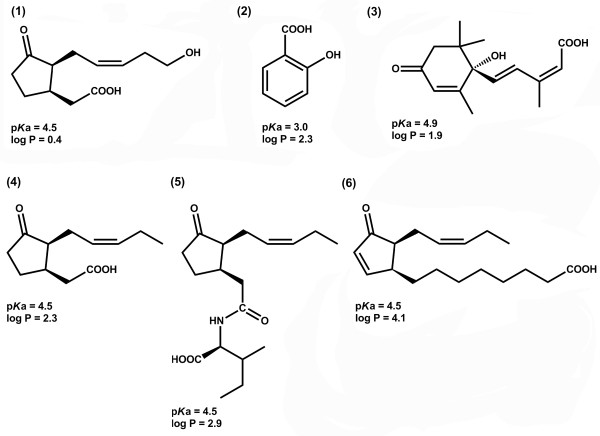
**The structures of target phytohormones: 12-hydroxyjasmonic acid, 12-OH-JA- (1); salicylic acid, SA (2); abscisic acid, ABA (3); (+)-7-*iso*-jasmonic acid, JA (4); (+)-7-*iso*-jasmonoyl isoleucine, JA-Ile (5); *cis*-12-oxophytodienoic acid, OPDA (6).**

Plant hormones play a key role in the regulation of plant development and response to various biotic and abiotic stresses. Salicylic acid (SA), abscisic acid (ABA), jasmonic acid (JA) and related compounds (Figure 1) are known to participate in plant response against pathogens, herbivores and abiotic factors such as UV irradiation, ozone exposure, high and low temperature, osmotic stress and mechanical wounding [1-5].

Wounding is known to trigger multiple plant defense mechanisms, such as synthesis of proteinase inhibitors, phytoalexins and volatile attractants, all directly mediated by jasmonates [4,6] and influenced by SA [1] and ABA [3]. (+)-7-*iso*-JA is synthesized in chloroplasts and peroxisomes [5] and is then converted into the biologically active conjugate (+)-7-*iso*-JA-isoleucine (JA-Ile) [2]. In *Arabidopsis thaliana*, JA accumulates locally in leaves already 30 s after wounding, and less than 5 min are necessary for a significant increase in JA-Ile levels [7]. The rise in JA and JA-Ile is accompanied by a rise of *cis*-12-oxophytodienoic acid (OPDA) in local and systemic leaves, respectively [8]. However, the fine mechanisms underlying distribution of JA, JA-Ile and their precursor OPDA as well as their hydroxylated and carboxylated derivatives [9] within plant tissues are still unknown. To fill this gap, highly-sensitive analytical approaches providing hormone content information for small (≤ 50 mg) tissue amounts are required.

Gas chromatography (GC) coupled on-line to mass spectrometry (MS) is routinely applied for the simultaneous analysis of multiple hormones in plant tissues [10]. However, as high analyte volatility is the prerequisite for proper GC-separation, derivatization of hydrophilic groups is required. Besides this relatively time-consuming step, even the modern GC-MS approaches still require at least 300 mg fresh plant material to reach dynamic range of a mass spectrometer [11]. Moreover, in order to reduce signal interference in quadrupole (Q) and ion trap (IT) mass analyzers, additional (most often multistep) depletion of non-hormone compounds is usually required [12]. These two factors dramatically reduce method sensitivity and sample throughput.

Reversed phase-high performance liquid chromatography (RP-HPLC)-electrospray ionization (ESI)-MS is an elegant way to overcome these limitations. High separation efficiency and selectivity of RP-HPLC minimizes ESI matrix effects [13], ensuring sensitive detection of analytes without additional derivatization procedures [9]. Tandem mass spectrometry (MS/MS) with triple quadrupole (QqQ) instruments operating in multiple reaction monitoring (MRM) mode is characterized with favorable duty cycles and reduced chemical noise that provides further sensitivity improvement [14]. Relatively short dwell times (≤ 50 ms) typical for modern QqQ mass spectrometers make a rapid analysis of multiple hormones possible [15]. Application of ultra performance liquid chromatography (UPLC) [16] as well as solid-phase extraction (SPE)-based enrichment [17] may further improve sensitivity of phytohormone analysis. Nevertheless, obtained in the presence of biological matrix method quantification limits (LOQ_m_) reported for JA, SA and ABA typically do not exceed 2.5 × 10^-14^ mol (even with top-sensitive instruments). Hence, relatively large sample amounts are still required for analysis.

Here we present a new sensitive, precise and high-throughput UPLC-ESI-MS/MS-based method for simultaneous analysis of jasmonates, SA, and ABA in plant tissues. After introduction of an SPE-based enrichment/depletion procedure and adjustment of both chromatographic and mass spectrometric parameters, phytohormones could be detected in plant extracts with LOQs of 10^-17^ - 10^-15^ mol. The procedure proved to be fast, universal, reproducible, and provided a throughput of at least 96 samples per day. Finally, the method was successfully applied to the study of wound-induced rise in jasmonates in tomato leaves (*Solanum lycopersicum*, cv. MicroTom), cross-validated with a well-established GC-MS procedure [12] and applied to various plant organs and species.

## Results and discussion

### Selection of quadrupole mass ranges for MS/MS analysis

In the initial step, SA, ABA, OPDA, JA, JA-Ile and 12-hydroxy-JA (12-OH-JA) were annotated in chromatograms of authentic standard mixtures by their retention times, *m*/*z* values of corresponding [M-H]^-^ ions and characteristic MS/MS fragmentation patterns (Additional file 1: Table S1). On the basis of this information, compound-dependent parameters, namely collision, declustering, collision cell entrance and exit potentials (CE, DP, CEP and CXP, respectively) were adjusted for selected precursor-fragment pairs by syringe pump infusion of 0.5 – 1.0 mmol/L solutions of individual authentic standards using Analyst 5.1 software (Additional file 1: Table S1). At least two Q1/Q3 mass range combinations (MRM-transitions) were selected for each compound. Thus, each analyte was characterized by two specific precursor-fragment ion combinations and a characteristic retention time (t_R_). For each analyte, quantification relied on the most intense transition (shown bold in Additional file 1: Table S1), while the less intense one was used to confirm the phytohormone assignment. The transitions, selected here for quantification (225/59, 137/93, 263/153, 209/59, 322/130, 291/165 for 12-OH-JA, SA, ABA, JA, JA-Ile and OPDA, respectively) were shown to yield the most abundant MS-signals also in other studies [14,18,19]. In order to correct phytohormone content values for effects related to the plant matrix and for losses during sample preparation, for all analytes we employed internal standardization using corresponding stable isotope-labeled counterparts amended to the extraction solvent. For these compounds (^2^H_6_-SA, ^2^H_6_-ABA, ^2^H_6_-JA, ^2^H_2_-JA-Ile and ^2^H_5_-OPDA), the Q1 and Q3 mass ranges selected for authentic standards were corrected for the presence of deuterium atoms (Additional file 1: Table S1) while the same set of MS parameters (CE, DP, CEP and CXP) was applied for their quantification as for the non-labeled isotopologues. As no structure-specific internal standard for 12-OH-JA was available, this compound was standardized by ^2^H_6_-JA.

### Optimization of chromatography system

The choice of an adequate chromatography system is the most important factor influencing sensitivity of LC-MS analysis. As all analytes presented in Figure 1 contain hydrophobic moieties in their structure, we have chosen RP-HPLC for their separation. In order to increase separation efficiency and, hence, sensitivity of chromatographic analysis while minimizing analysis time, we applied a C18 reversed phase column filled with 1.8 μm particles and UPLC instrumentation. Special attention was paid to the mobile phase composition (i.e. organic modifier and additives), as it directly influences both LC-separation and MS-detection [20].

For sufficient retention of acids on reversed phase their protonated state is a prerequisite [21], while basic (i.e. deprotonating) additives enhance ionization in an ESI source operating in negative ion mode [22]. All previous eluent selection strategies relied either on acidic (formic and acetic acids) [15,18] or basic (ammonium acetate) [23] additives favoring only separation or ionization, respectively. Notably, some polar carboxylated phytohormones (e.g. SA) are partly dissociated at pH 4.0. Thus, their complete retention on C18 reversed phases in presence of ammonium acetate buffers was impaired (Figure 1). We assumed that the use of ammonium formate buffer adjusted with formic acid to pH 3.5 would improve both chromatographic and ionization behavior of acidic phytohormones. Therefore, the effect of 0.3 mmol/L formic acid (pH 2.7), 0.3 mmol/L ammonium acetate (pH 4.0) and 0.3 mmol/L ammonium formate (pH 3.5) on intensities of SA [M-H]^-^ ions was compared. Indeed, 0.3 mmol/L ammonium formate resulted in highest signal intensities in Q1 scans (Additional file 1: Figure S1). Reducing or increase of buffer ionic strength did not result in further improvements in ionization efficiency (Additional file 1: Figure S2). Thus, overall 10 and 25% increase in intensity of phytohormone [M-H]^-^ ions in comparison to typically used formic acid- and ammonium acetate-based systems was achieved.

**Figure 2:**
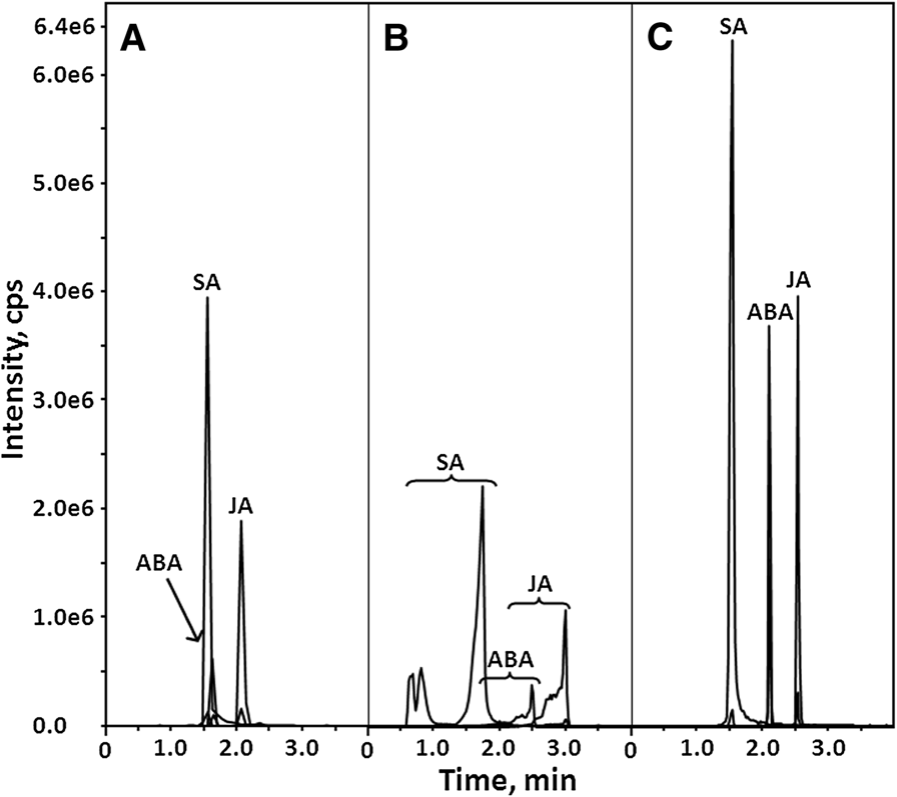
**Reversed phase chromatograms of a standard mixture containing 1 μmol/L SA, ABA and JA in 20% aq. methanol (injection volume of 1 μL) acquired in water-acetonitrile eluent system containing 0.1% formic acid (A), 0.3 mmol/L ammonium acetate (B) or 0.3 mmol/L ammonium formate (C).** The reversed phase chromatograms were recorded by a QqLIT mass spectrometer operating in negative MS/MS (MRM) mode.

Further, the effect of three buffer additives on retention and separation of acidic phytohormones on reversed phase was studied with the mixture of 1 μmol/L SA, ABA and JA. Although methanol is widely used as organic modifier in RP-HPLC-based phytohormone analysis [15,18,24,25], here we used only acetonitrile, because of its higher elution power and, hence, higher separation efficiency and sensitivity of analysis [26]. Although 0.1% formic acid (pH 2.7) provided good retention for all analytes, ABA and SA could not be completely separated under selected gradient conditions (Figure 2A). Increase of eluent pH to 4.0 (0.3 mmol/L ammonium acetate buffer) resulted in essential peak broadening and fronting, whereas use of ammonium formate buffer with pH 3.5 yielded optimal chromatographic performance (Figure 2B and C, respectively).

### Solid phase extraction

As phytohormones are present in plant tissues in the concentration range of 10^-9^ – 10^-6^ mol/L [17], strong enrichment is desired for their adequate quantification by mass spectrometry (especially if only small sample amounts are available). Previously, it was achieved by liquid-phase extraction (LPE) with dichloromethane or diethyl ether [18,27,28] or solid-phase extraction (SPE) using RP [15,17,25,29], anion [30,31] or cation exchange [32] materials. Besides enrichment, these procedures also provide a reduction in sample complexity, minimizing matrix effects during the ESI-process [13]. As most of the published applications rely on C18-RP-SPE, in our initial experiments we used cartridges filled with this type of material (Chromabond C18 ec, Macherey-Nagel, Düren, Germany). However, due to different pK_S_ or the logP values (Figure 1), the analytes varied strongly in their solubility in aqueous methanolic mixtures and demonstrated different retention behavior on C18 material. Thus, it was impossible to achieve good recovery for all phytohormones in the analyzed standard mixtures (Additional file 1: Figure S3). Even application of 10% aq. methanol (a concentration of organic modifier necessary for quantitative dissolving of all analytes) as a loading solvent for a mixture of JA, ABA and SA, did not ensure efficient retention of the most hydrophilic component SA. Its loss in the wash fraction was essential (Additional file 1: Figure S3). In contrast, ABA, a more hydrophobic analyte, remained partly retained even after elution with acetonitrile (Additional file 1: Figure S3). Such a behavior of phytohormones on C18 SPE material was not surprising as the restricted applicability of silica-based C18 material for simultaneous enrichment of multiple phytohormones was previously reported [18]. Consequently, SPE procedures based on combination of several (at least two) separation principals, suitable for retention of both hydrophilic and hydrophobic compounds, were expected to provide sufficient retention of all analytes.

**Figure 3:**
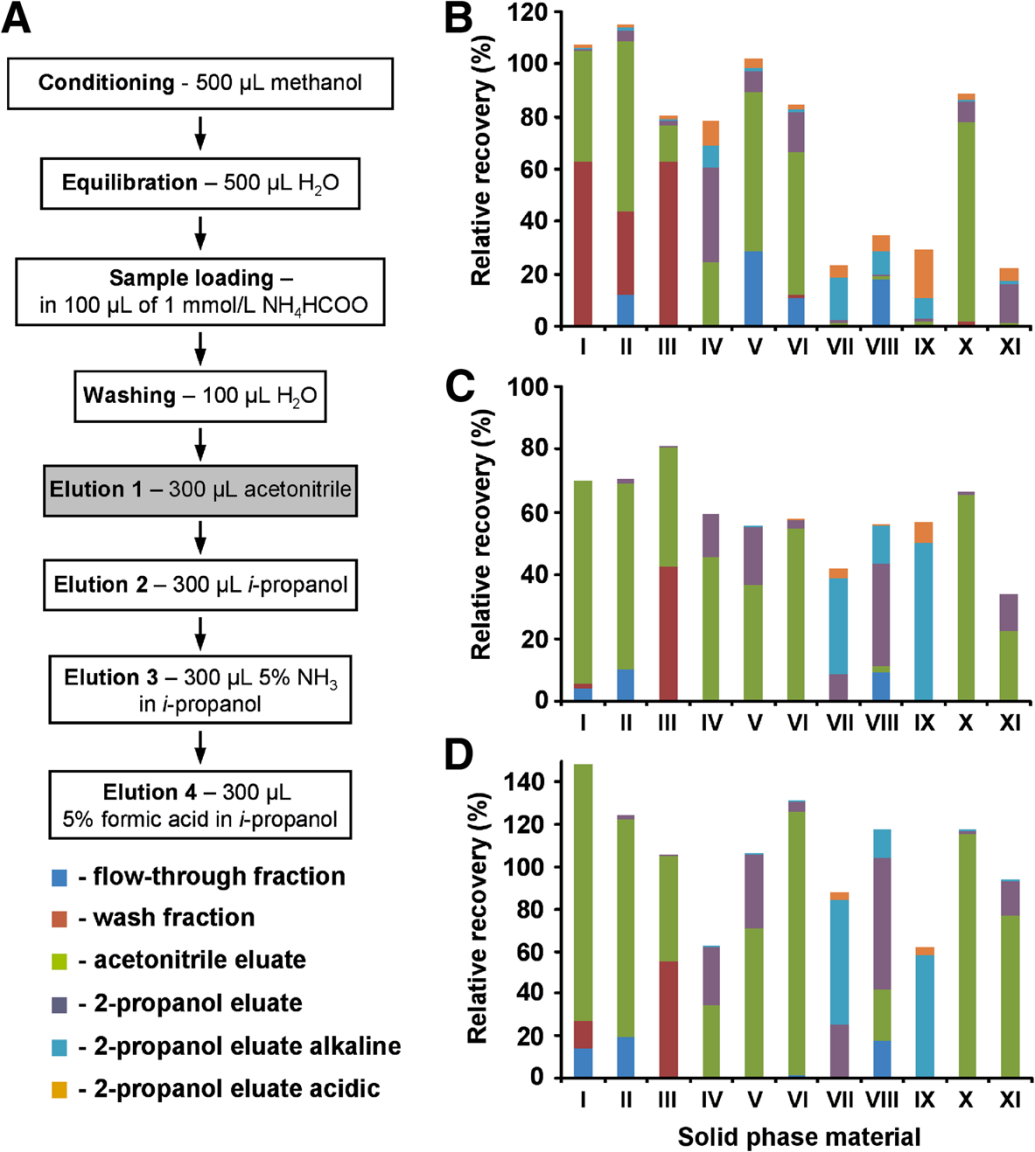
**Extraction protocol (A) and recoveries of SA (B), ABA (C) and JA (D) in flow-through (blue), wash (red), acetonitrile elution (green), isopropanol elution (violet), alkaline elution (sky-blue) and acidic elution (orange) fractions observed after loading of a standard mixture containing 19.8 mg/L of SA, ABA and JA (100 μL) on solid phase material of different specificity: Chromabond C18ec (I), Strata C18-E (II), Spec C18 AR (III), Chromabond HR-X (IV), OASIS HLB (V), Bond Elut PLEXA (VI), Strata X-AW30u (VII), OASIS WAX (VIII), Chromabond HR-XAW (IX), OASIS MCX (X), OASIS WCX (XI).** Measurements were performed in triplicates.

In order to select an appropriate phase capable of retaining analytes within a broad range of hydrophobicity and pKa values (Figure 1), a screening experiment with eleven commercially available solid phase materials possessing different chemistry and specificity was performed using a standard mixture of three hormones – SA, ABA and JA. To reduce sample preparation time (i.e. to increase sample throughput), we switched to 96-well plate scale while performing all SPE steps in a centrifuge at low speed. Among the SPE materials tested we employed three silica-based and two polymer-based reversed phase materials, as well as one polar-functionalized, three anion- and two cation-exchange polymer-based resins (Additional file 1: Table S2). Five ion exchange materials relied on a dual retention mechanism based on hydrophobic interactions with a polymeric backbone in addition to ion exchange on charged surfaces, i.e. so called “mixed mode” phases. To provide a reliable comparison of the analyte recovery from different SPE-materials, four consecutive elution steps were employed to disrupt hydrophobic, anionic and cationic interactions of analytes with the solid phases (Figure 3A). Flow-through, wash and four eluate fractions were collected, and 3.3 μL of each effluent was subjected to RP-UPLC.

Analysis of the fractions revealed essential break-through of all three phytohormones upon sample loading and washing when silica-based C18 reversed phase materials were applied (Figure 3B – D, I – III). Moreover, in contrast to other phases, duration of solvent filtration through cartridge material was irreproducible. It led to lower recovery precision due to drying of the C18 material (Additional file 1: Table S3). As can be seen from Figure 3B – D (I – V), most of the loaded analyte retained on reversed phase was recovered with acetonitrile in the first elution step (Additional file 1: Figure S4), whereby polymer-based RP materials showed lower losses during loading and washing steps. However, much stronger retention of all analytes on the latter materials (IV – V) and polar-functionalized phase (VI) was observed: even a stronger eluent (isopropanol) did not result in quantitative elution of abscisic acid from polymer-based reversed phases (Figure 3C).

In contrast, all three tested weak anion-exchange resins (VII-IX) as well as the weak cation-exchange resin (XI) provided reduced recoveries for SA and ABA (Figure 3B – C). For weak anion exchange resins it could be explained by a strong break-through in flow-through and wash fractions (Figure 3B – C). In contrast, extremely high retention even for less hydrophobic analytes was observed on the weak cation exchange material (XI): SA, as the strongest acid among the three analytes, resulted in only 24.2% recovery in all eluate fractions (Figure 3B, Additional file 1: Table S3, ). Most likely, a reversed phase mechanism played essential role in this phenomenon, as elution under alkaline conditions was negligible (Figure 3). It is important to note, that recoveries in acetonitrile fraction were, however, highly reproducible for most of the phases: RSD% values did not exceed 16% for any of analytes and were typically less than 10% (Additional file 1: Table S3).

While relatively good analyte recoveries from weak anion exchange material during alkaline elution is supported by discharge of weak bases at the solid phase surface, high retention of anionic phytohormones on a strong cation exchanger was unexpected. However, due to the dual chemistry of this phase, relatively hydrophobic phytohormones were better retained by a RP mechanism. Additionally, an exchange of cationic metabolites (i.e. counter-ions) present in plant extracts against surface bound protons (H-form) may shift acid–base equilibrium of carboxylated hormones towards the non-dissociated forms, thus enhancing hydrophobic interactions of also relatively polar hormones with polymeric material.

Besides, resulted from ion exchange free protons will only temporarily decrease the pH since they are washed out upon loading whereas retarded matrix cations are not eluted from the solid phase with base-free acetonitrile. Thereby, depletion of the plant extract for cationic and zwitter-ionic metabolites could be achieved. Possibly, pre-load acidification of hormone extracts would result in better retention of phytohormones on RP materials. However, as a pH decrease might influence hormone stability, use of cation exchange SPE seems to be favorable. Not less important, exchange of matrix cations reduces sample complexity diminishing thereby possible matrix effects during electrospray ionization.

On the basis of the obtained results, strong cation exchange polystyrene/ divinylbenzene (PS/DVB)-resin was selected for further experiments. In order to reduce analysis costs, for further analyses we switched to PS/DVB-based Cromabond® HR-XC material (Macherey-Nagel, Düren, Germany), a close analog to the OASIS MCX phase. High recoveries from this phase were confirmed for all six phytohormones of interest in additional experiments (see below).

### Method validation

Due to improved mobile phase composition, the LC-MS/MS method demonstrated remarkable sensitivity: the instrument limits of detection and quantification (LOD_i_ and LOQ_i_, respectively) were between 25 amol for JA-Ile and 2.5 fmol for 12-OH-JA (Additional file 1: Table S4). That is, to the best of our knowledge, superior in comparison to the most of published methods [17,18,25,33,34]. Recently, Liu and collaborators reported similar method sensitivity parameters [35]. However, the authors employed a much more sensitive (and, hence, expensive) mass spectrometer. In our study, all analytes demonstrated high linear dynamic ranges (LDRs) – typically more than 1 x 10^3^ (only for 12-OH-JA this value was 4 x 10^2^), with R^2^ > 0.99 (Additional file 1: Table S4). The high sensitivity can be explained by employment of UPLC technique [36], providing superior efficiencies of separation and, hence, typical peak widths (w_1/2_) of several seconds. However, due to pronounced matrix effects produced by multiple components of plant extracts, the limits of quantification (LOQ_m_) were expected to be essentially higher.

To determine the analyte LOQ_m_ values in a representative plant matrix, a standard addition approach was employed. For this, methanolic plant extracts obtained from 20 mg of fresh material were spiked with a mixture of stable isotope-labeled phytohormone standards prior to SPE enrichment and LC-MS/MS analysis. Assuming the same analytical response (i.e. the same ionization efficiencies and fragmentation patterns) for these stable isotope-labeled compounds as for their natural counterparts, the LOQ_m_ values might correspond well to those for the native plant hormones. All together, eight concentration levels of plant hormones were applied. The LOQ_m_ values were determined as amounts or contents (in fmol or ng/mg, respectively) corresponding to the lowest analyte signal/noise ratios yielding significantly higher response in comparison to a next lower concentration level when tested by the paired t-test at 0.05 error probability [37]. The observed LOQ_m_ values were in lower femtomole range (Table 1) being superior in comparison to other data obtained with the standard addition method [7,19].

**Table 1:** **Method limits of quantification (LOQ**_**m**_**) determined in complex plant extracts**

**Stable isotope labeled standards**^**a**^	**LOQ, nmol/L**	**LOQ, nmol/L, concentrated sample**^**b**^
^2^H_6_-SA	5.80	0.58
^2^H_6_-ABA	12.93	2.34
^2^H_6_-JA	12.93	1.81
^2^H_2_-(−)-JA-Ile	1.73	0.22
^2^H_5_-OPDA	1.89	0.34

^**a**^The standard mixture was added at eight concentration levels (0.14 – 27.33 μg/L, n = 4) to 500 μL of plant extract obtained from 20 mg of the mixture of rice leaf, rice root, tomato leaf, tomato root, potato leaf and *Arabidopsis* leaf. The spiked extracts were subjected to SPE and LC-MS/MS analysis as described in Methods part with and without subsequent drying/reconstitution step. 3.3 μL of SPE eluate was injected in UPLC.

^**b**^ The SPE eluates were dried completely under nitrogen stream and reconstituted in 40 μL acetonitrile. After addition of 40 μL 0.3 mmol/L aq. ammonium formate, the samples were transferred to glass vials and 3.3 μL of solution was injected in UPLC.

In order to further increase the method sensitivity, the eluate SPE fractions were completely dried and reconstituted in small volumes of solvent. Among three tested approaches, drying under nitrogen flow yielded the highest recoveries while 50% aq. acetonitrile proved to be the most suitable solvent for sample reconstitution, providing at least 70% recovery for all analytes (Additional file 1: Table S5). These results were in accordance with the relatively high hydrophobicity of OPDA (Figure 1). This analyte could not be completely dissolved in aqueous solutions with low organic content. Therefore, the dried samples were first reconstituted in acetonitrile and diluted afterwards in a 1:1 (v/v) ratio with 0.3 mmol/L aq. NH_4_HCOO. This procedure provided an additional 5.5 – 10.0-fold reduction of LOQ_m_ values (Table 1). Thus, the method presented here can be successfully applied to phytohormone quantification in tissue samples of less than 50 mg. This reduction of sample amount would provide a possibility for characterization of phytohormone gradients in plant organs, i.e. to get a deeper understanding of the mechanisms of hormone-mediated signaling.

The recoveries of all six phytohormones were determined by a standard addition method. For this, SA, ABA, JA, 12-OH-JA, JA-Ile and OPDA were diluted in methanol to the concentrations of 0, 1.3, 2.5, 5.0 and 10 μg/L. 500 μL of these standard mixtures were used for extraction in presence and absence of complex plant matrix and subjected afterwards to RP-UPLC-MS/MS with and without SPE enrichment (see Methods). The analyte abundances (peak heights) were plotted against their corresponding concentrations and linear regression parameters were derived (Table 2). The phytohormone recoveries were determined as slope ratios obtained from concentration-intensity plots based on the data acquired with and without SPE (Table 2). All analytes showed high recoveries (88% or more) irrespective of the interactions with components of plant extract or matrix effects during electrospray ionization. However, as the extracts were further concentrated via a drying/reconstitution procedure, recovery of all hormones besides SA declined. Moreover, lower R^2^ values for concentration-analyte intensity plots obtained for dried/reconstituted standard mixtures indicated distinctly lower precision of this strategy. These losses may be, however, corrected by the use of stable isotope-labeled internal standards. This approach would further decrease the sample amount required for the hormone assessment. However, as desired sensitivity and precision was achieved here without sample drying/reconstitution, this variant of the method was not further investigated.

**Table 2:** Recovery of phytohormones from different sample matrices on polystyrene/ divinylbenzene-based Cromabond® HR-XC material

**Matrix**	**SA**			**ABA**			**JA**			**12-OH-JA**			**JA-Ile**			**OPDA**		
	**Slope**	**R**^**2**^	**Rec.**	**Slope**	**R**^**2**^	**Rec.**	**Slope**	**R**^**2**^	**Rec.**	**Slope**	**R**^**2**^	**Rec.**	**Slope**	**R**^**2**^	**Rec.**	**Slope**	**R**^**2**^	**Rec.**
**Recovery control**	453.48	0.99	***1.00***	75.12	0.99	***1.00***	105.27	0.99	***1.00***	431.91	0.99	***1.00***	541.36	0.99	***1.00***	126.64	0.99	***1.00***
**20% Methanol**	397.59	0.99	**0.88**	90.73	0.99	**1.21**	110.37	0.97	**1.05**	497.68	1.00	**1.15**	533.24	1.00	**0.98**	122.33	1.00	**0.97**
**Methanolic extract**	558.56	0.96	**1.23**	76.50	0.92	**1.02**	108.49	0.97	**1.03**	444.99	0.98	**1.03**	532.82	1.00	**0.98**	118.41	0.99	**0.93**
**Reconstituted methanolic extract**	392.81	0.77	**0.86**	55.25	0.86	**0.49**	81.27	0.88	**0.64**	156.24	0.83	**0.44**	424.25	0.99	**0.67**	77.65	0.95	**0.50**

The analyte abundances (peak heights) were plotted against their defined concentrations and correlation of these two parameters was estimated (linear regression coefficient was calculated). The phytohormone recoveries (Rec.) were determined as ratios of slopes compairing corresponding concentration-intensity plots obtained with and without SPE procedure.

### Study of wounding kinetics in solanum lycopersicum

In the next step the designed method was verified by recording the levels of jasmonates in mechanically wounded leaves of tomato (*Solanum lycopersicum*, cv. MicroTom). To elucidate the kinetics of OPDA, JA and JA-Ile, methanolic extracts were obtained in triplicates from leaves at different time points after wounding and were spiked with the mixture of internal standards as described in Methods. The individual extracts were then split in two aliquots, one of which was analyzed by the novel LC-MS/MS method, while the second one was subjected to a well-established GC-MS procedure in a cross-validation experiment [12].

**Figure 4:**
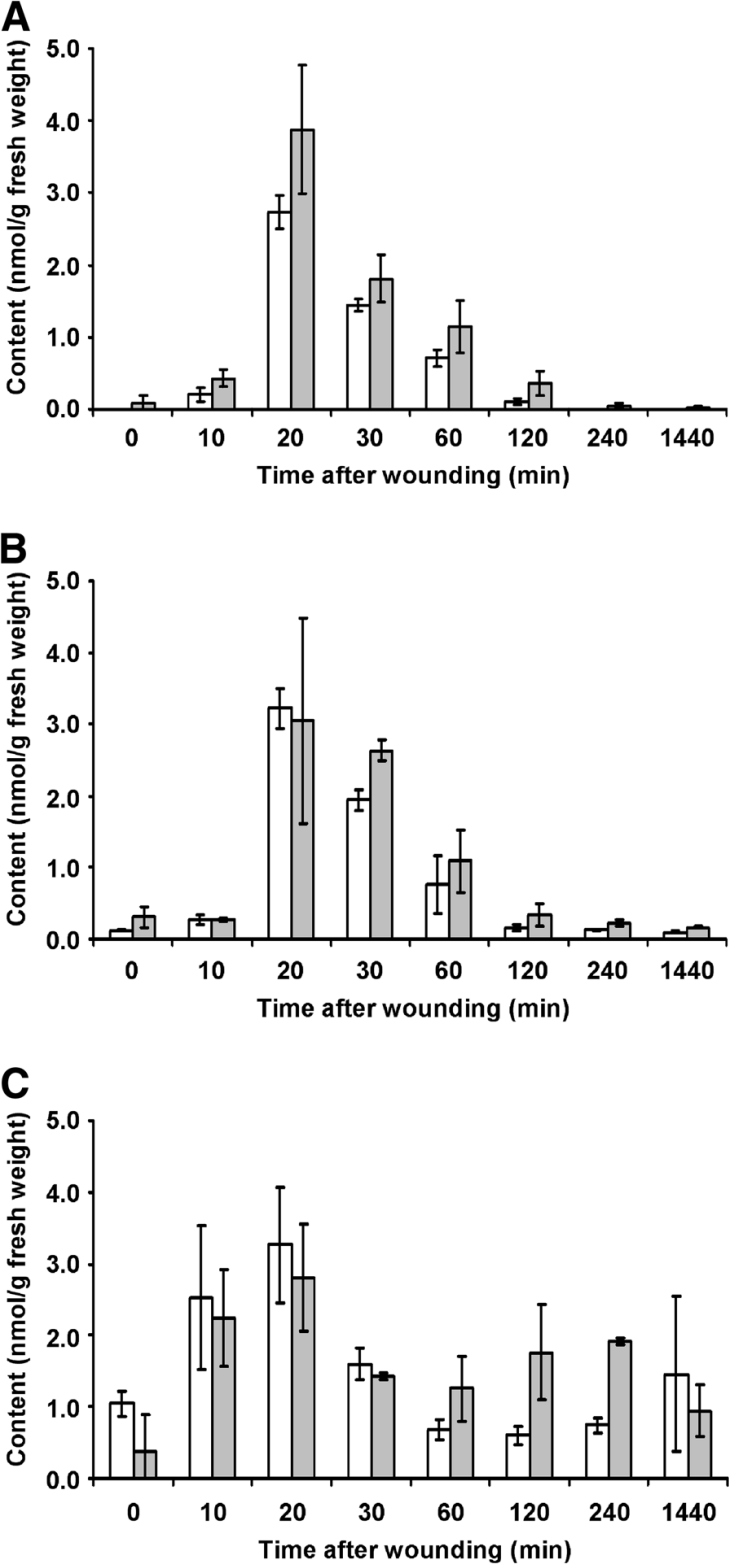
**Dynamics of JA (A), JA-Ile (B) and OPDA (C) content in mechanically wounded leaves of tomato (*Solanum lycopersicum *cv. MicroTom).** The contents were determined in triplicates with the new LC-MS/MS method (white columns) and the previously established GC-MS one (gray columns) before and at 10, 20, 30, 60, 120, 240 and 1440 min after wounding.

Mechanical wounding is one of the best-elucidated triggers of a local rise in jasmonate levels [5,6]. The dynamics of jasmonate contents in wounded tomato leaves is well-characterized. JA levels [31,38,39] increase already within minutes after wounding reaching the maximum 30 min after stress exposure. This increase is accompanied by accumulation of the physiologically active hormone JA-Ile, the contents of which, however, typically do not exceed the maximal levels detected for JA [9]. Also for the tomato cultivar MicroTom, the data mirror this pattern: the maximum contents of both JA and JA-Ile were detected at 20 min after wounding (Figure 4A and B). Differences in timing and height of these maxima in the different experiments (data shown in Figure 4*versus*[31]) might be due to differences in the tomato cultivar, the developmental stage of plants, the growth conditions and the strength of wounding. As expected, the content of the JA precursor OPDA was slightly increased already 10 min after wounding (Figure 4C). Most importantly, the results obtained with the new LC-MS/MS method were in agreement with the data obtained from the identical samples using GC-MS This demonstrates the accuracy of JA, JA-Ile and OPDA determination by the new method.

### Applicability of the method to other plant species and organs

In order to check applicability of the analytical approach presented here to other plant species, developmental stages and organs, the method was cross-validated with plant material of different origin: rice (*Oryza sativa*) roots, *Arabidopsis thaliana* seedlings and rosette leaves, *Medicago truncatula* roots and leaves. All the samples obtained all from wild type and non-stressed plants (50 mg fresh weight, n = 5) and were extracted as described for tomato leaf, subjected to SPE and the eluates were analyzed for phytohormone contents by RP-UPLC-MS/MS. The resulted values (Additional file 1: Table S6) were in accordance with published data [12,40-42]. Thus, the method was shown to be applicable for any plant object and can be used in any field of plant biology and biochemistry.

In summary, our method delivers reliable values for phytohormone contents in different plant species or plant organs, such as roots, stems, flowers, and seeds. In comparison to existing hormone quantification methods it provides superior sensitivity, precision, and linear dynamic ranges for six plant hormones tested. The resulted concentrations correspond well to those obtained in an independent cross-validation experiment with another analytical approach.

## Conclusions

Jasmonates, ABA and SA are the key players in the regulation of plant defense against pathogen and herbivore attack. Knowledge about their spatial and temporal distribution in plant tissues is necessary for understanding the mechanisms of local and systemic plant response to these biotic stress factors. However, for quantification of phytohormones highly sensitive and precise analytical methods are required. We have introduced a high-throughput LC-MS/MS-based approach for absolute quantification of four jasmonates, SA and ABA in methanolic extracts of plant tissue. In the sample preparation step, a quantitative enrichment of phytohormones and depletion of cationic metabolites and photosynthetic pigments was achieved on mixed-mode RP/cation exchange polymeric resins. Besides, LC mobile phase composition was adjusted to provide optimal chromatographic resolution and higher MS-ionization efficiency. Combined, these approaches resulted in a dramatic sensitivity increase in comparison to previously established methods. Due to this sensitivity gain, phytohormone analysis can be successfully performed with a wide range of commercially available mass spectrometers with a low plant material consumption. The validated method was successfully applied to quantitative characterization of jasmonate concentration dynamics in *Solanum lycopersicum* leaf tissues and cross-validated with a well-established GC-MS method.

## Methods

### Reagents

(±) Abscisic acid (ABA), salicylic acid (SA), ammonium acetate, ammonium formate, formic acid, acetonitrile and methanol (all LC-MS grade) were from Sigma Aldrich (Taufkrichen, Germany). JA was obtained by alkali hydrolysis of methyl jasmonate (MeJa, Firmenich, Geneva, Switzerland). JA-Ile was kindly provided by Dr. Kramell (IPB). 12-Hydroxyjasmonic acid (12-OH-JA) was obtained as described by Kitahara et al. [43]. OPDA was synthesized from linolenic acid using linseed enzyme [44]. ^2^H_6_-ABA was synthesized according to Gómez-Cadenas et al. [45]. ^2^H_6_-JA was obtained as described by Miersch et al. [46]. ^2^H_2_-(−)-JA-Ile was prepared from ^2^H_2_-(±)-JA and Ile [47]. ^2^H_5_-OPDA was prepared from (17-^2^H_2_, 18-^2^H_3_)-linolenic acid as described by Zimmerman and Feng [44]. ^2^H_6_-SA was purchased from Campro Scientific (Veenendaal, Netherlands). Water was purified in-house by a Milli-Q Plus Ultrapure Water System (Millipore GmbH, Schwalbach, Germany) (resistance >18 MΩ).

### Extraction of phytohormones from plant tissues

20 – 50 mg of fresh plant material was homogenized in a mortar under liquid nitrogen and extracted with 500 μL methanol containing 0.1 ng/μL of each stable isotope-labeled internal standard (^2^H_6_-SA, ^2^H_6_-ABA, ^2^H_6_-JA, ^2^H_2_-(−)-JA-Ile and ^2^H_5_-OPDA). The extraction was performed in 1.6 mL cryo-tubes (Precellys Steel Kit 2.8 mm, Peqlab Biotechnologie GmbH, Erlangen, Germany) using a bead mill (FastPrep24 instrument, MP Biomedicals LLC, Santa Ana, CA, USA) with acceleration of 6.5 m/s^2^ for 30 s. After centrifugation at 20000 g (2 min, 0°C), 450 μL of supernatant was transferred into a polypropylene tube, diluted with water to 5 mL and subjected to solid-phase extraction (SPE).

### Solid-phase extraction (SPE)

Solid phase extraction was performed in a 96-well plate format using filter plates and deep well receiving plates in conjunction with centrifugation. For the phase screening experiments, 96-deep well filter plates (Agilent Technologies, Böblingen, Germany) were packed with 15 – 60 mg of SPE materials representing different chemistry and specificity (Additional file 1: Table S2). For the solid phase screening experiment, the plates were conditioned with 500 μL methanol and equilibrated with 500 μL of water before loading of the standard mixture containing 18.8 mg/L SA, ABA and JA dissolved in 1 mmol/L ammonium formate. After washing with 100 μL of water, phytohormones were consecutively eluted by 300 μL portions of acetonitrile, isopropanol, 5% aq. NH_4_OH in isopropanol, and 5% formic acid in isopropanol. All steps were performed by centrifugation at 10 g (Avanti J-E centrifuge, Beckman-Coulter, Kreefeld, Germany). The fractions were collected in separate tubes and stored at −20°C.

For analysis of plant samples and calibration mixtures, the same 96-well filter plates were packed with 50 mg of a strong cation exchange HR-XC material (Macherey & Nagel, Düren, Germany). The material was conditioned with 1 mL methanol and equilibrated with 1 mL water. Afterwards, the plant extracts, diluted as described previously, were loaded on each well in five 1 mL-portions. The fraction containing phytohormones was eluted by 900 μL acetonitrile without additional washing. 850 μL of the eluate fraction were transferred to a 1.5-mL polypropylene tube and storied at −20°C before LC-MS analysis. Alternatively, eluate fractions were completely dried before storage.

### RP-UPLC-ESI-MS/MS

Separations were performed on a Waters HSS T3 C18 column (1 x 100 mm, particle size 1.8 μm) at 40°C using a Waters ACQUITY UPLC System, equipped with an ACQUITY Binary Solvent Manager and ACQUITY Sample Manager (20 μL sample loop, partial loop injection mode, 3.3 μL injection volume). Eluents A and B were water and 90% aq. acetonitrile, respectively, both containing 0.1% HCOOH, 0.3 mmol/L NH_4_CH_3_COO (adjusted to pH 4.0 with acetic acid), or 0.3 mmol/L NH_4_HCOO (adjusted to pH 3.5 with formic acid). Elution was performed isocratically for 0.5 min at 5% eluent B and then consecutive linear gradients to 30, 80 and 95% eluent B in 5, 0.5 and 2 min, respectively were run. The column was re-equilibrated for 3 min. The flow rate was set to 150 μL/min and the column temperature was maintained at 40°C. Phytohormones were detected on-line by ESI-MS/MS using a 3200 Q TRAP® LC/MS/MS System hybrid QqLIT mass spectrometer equipped with an ESI-TurboIon-Spray™ interface, operating in negative ion mode and controlled by Analyst 1.5 software (AB Sciex, Darmstadt, Germany). The LC-ESI source operation parameters were as the following: ion spray voltage, -2700 V; nebulizing gas, 40 psi; source temperature, 550°C; drying gas, 40 psi; curtain gas, 25 psi. Instrument tuning and mass calibration were performed with 100 μmol/L polypropylene glycol solutions.

QqQ scans were acquired as multiple reaction monitoring (MRM) experiments with Q1 and Q3 resolution set as “unit”. Dwell times were defined by “scheduled MRM” function set as following: MRM detection window, 70 s and targeted scan time, 1 s. Compound-dependent parameters for authentic standards were optimized individually in flow injection experiments and further applied to their stable isotope-labeled counterparts (summarized in Additional file 1: Table S1, ). For these measurements, the ESI source operation parameters were set as following: ion spray voltage, -4500 V; nebulizing gas, 30 psi; source temperature, room temperature (RT); drying gas, 0 psi; curtain gas, 10 psi.

For infusion experiments, a syringe pump (Hamilton OEM Syringe Pump, 10 mL Syringe, Hamilton Bonaduz AG , Bodanuz, Switzerland) operating at 10 μL/min was coupled on-line to the same ESI-QqLIT mass spectrometer operating in negative ion mode. Individual analytes were dissolved in aq. formic acid (pH 2.7), ammonium acetate and ammonium formate buffers (adjusted to pH 4.0 and 3.5 with acetic and formic acids, respectively) at the concentration of 1 μmol/L. The concentrations of solvent additives ranged from 0.1 to 3.0 mmol/L. The analytes were tracked in Q1 scans by their [M-H]^-^ ions.

### Method validation

For external calibration and instrument detection (LOD_i_) and quantification (LOQ_i_) limit determination, the mixture containing 1 mmol/L of each individual standard was serially diluted with 20% aq. methanol by 2.0 – 2.5-fold increment to obtain 23 concentration steps (0.01 nmol/L – 500 μmol/L). The method limits of quantification (LOQ_m_) were determined by the standard addition method [37]. For this, 20 mg of lyophilized plant pool (i.e. a mixture consisting of tomato leaves and roots, potato leaves, rice leaves and roots, barley leaves and *Arabidopsis* leaves mixed in equal proportion) was spiked with a standard mixture containing ^2^H_6_-SA, ^2^H_6_-ABA, ^2^H_6_-JA, ^2^H_2_-(−)-JA-Ile, ^2^H_5_-OPDA at eight concentration levels (0.14 – 27.33 μg/L). The spiked samples were extracted with methanol and subjected to SPE as described above.

Recoveries of phytohormones on PS/DVB-based Cromabond® HR-XC material (Macherey & Nagel, Düren, Germany) were determined by the standard addition method. SA, ABA, JA, 12-OH-JA, JA-Ile and OPDA were diluted in 500 μL aliquots of methanol at the concentrations of 0, 1.3, 2.5, 5.0, 10.0 and 15 μg/L and processed with and without extraction of complex lyophilized plant pool (20 mg). The obtained extracts were subjected to RP-LC-MS/MS with and without further drying in nitrogen stream. The residues were reconstituted in 40 μL acetonitrile before further dilution to 80 μl with 0.3 mmol/L aq. ammonium formate (pH 3.5).

The analyte abundances expressed as peak heights were plotted against their concentrations. Additionally, regression coefficients and R^2^ values were calculated.

### Plant material and wounding experiments

Seeds of tomato plants (*Solanum lycopersicon* cv. MicroTom) were germinated on expanded clay of 2–5 mm particle size (Original Lamstedt Ton; Fibo ExClay, http://www.fiboexclay.de) watered with tape water. After one week, seedlings were transferred into soil, grown in a phytotron at 29°C, 50% relative humidity and 18-h light/6-h dark cycle, and were watered every second day with tape water. *Oryza sativa* L., *Arabidopsis thaliana* (L.) Heynh and *Medicago truncatula* Gaertn. plants were grown as described elsewhere [12,40-42]. Wounding of tomato plants was performed at onset of flowering (about six weeks after sowing) and was done by squeezing the leaflets of two leaves per plant five times across the mid-vein with tweezers. For each point a separate plant was used. The tomato leaves (two leaves per plant only) were harvested before wounding and 10, 20, 30, 60, 120, 240 and 1440 min post-treatment. Tissue extraction, SPE and RP-HPLC-MS/MS were performed as described above. The organs obtained from unstressed plants of other species (*O*. *sativa* roots, *A*. *thaliana* seedlings and rosette leaf, *M*. *truncatula* roots and leaf) were treated in the same way. The content of individual phytohormones was calculated as the ratio of analyte and internal standard peak heights multiplied by the concentration of the corresponding internal standard.

### Quantitative determinations of OPDA, JA and JA-Ile by GC-MS

Methanolic extracts obtained from wounded tomato leaves were subjected to the well established GC-MS-based strategy comprising ion exchange chromatography and RP-HPLC purification steps [12].

## Competing interests

The authors declare that they have no competing interests

## Authors’ contributions

GB, VH, BH and AF designed the experiments; AH and HS performed sample preparation; VH, NB and MF performed UPLC-MS/MS analyses; GB, VH, AT, BH and AF were involved in writing the manuscript. All authors read and approve the final manuscript.

## Supplementary Material

Additional file 1 Supplementary information. Click here for file
