# Supplementary material for: An UPLC-MS/MS method for highly sensitive high-throughput analysis of phytohormones in plant tissues
Source: Plant Methods. 2012 Nov 22;8:47. doi: 10.1186/1746-4811-8-47 (PMC3573895; doi:10.1186/1746-4811-8-47)
Supplement: Additional file 1 — Supplementary information. [file 1746-4811-8-47-S1.doc]

An UPLC-MS/MS method for highly sensitive high-throughput analysis of phytohormones in plant tissues

Gerd Ulrich Balcke^†^, Vinzenz Handrick^†‡^, Nick Bergau, Mandy Fichtner, Anja Henning, Hagen Stellmach, Alain Tissier, Bettina Hause and Andrej Frolov^*^

**Additional file 1**

Leibniz Institute of Plant Biochemistry, Department of Cell and Metabolic Biology, Weinberg 3, 06120 Halle (Saale), Germany

^†^These authors contributed equally to the work

^‡^Present address: Max Planck Institute for Chemical Ecology, Department of Biochemistry

Hans-Knoell-Str. 8, 07745 Jena, Germany

*Corresponding author

Dr. Andrej Frolov

Present address:

Leipzig University

Faculty of Chemistry and Mineralogy

Institute of Bioanalytical Chemistry

Centre for Biotechnology and Biomedicine

Deutscher Platz 5

04103 Leipzig

Germany

Tel. +49 (0) 341 9731332

Fax. +49 (0) 341 9731339

E-mail: [andrej.frolov@bbz.uni-leipzig.de](mailto:andrej.frolov@bbz.uni-leipzig.de)

**Table A-1** Annotation of individual phytohormones and their stable isotope-labeled counterparts by t_R_, *m/z* values and their identification by MS/MS fragmentation patterns.

| # | **Substance^a^** | **t_R_, min** | **Pseudo-molecular ion, *m/z*** | | **Fragment ions (*m/z*) observed in Enhanced Product Ion (MS/MS) scan, relative intensity (%) ^b,c,d^** | **Optimalg**  **MRM- transition(s)^e^** | **Collision potential**  **(CE), V** | **Declustering potential (DP), V** | **Entrance potential (EP), V** | **Cell exit potential (CXP), V** |
| --- | --- | --- | --- | --- | --- | --- | --- | --- | --- | --- |
| 1 | 12-Hydroxyjasmonic acid | 1.6 | [M-H]^-^ | 225.0 | 59 (100), 97 (7), 135 (7), 148 (6), 151 (3), 165 (4), 181 (2), 207 (2), 225 (97) | **225 → 59**  225 → 147 | -24  -16 | -38  -40 | -20  -20 | -4  -4 |
| 2 | Salicylic acid | 1.7 | [M-H]^-^ | 136.9 | 65 (2), 93 (100), 137 (70) | **137 → 93**  137 → 75 | | -20 | | --- | | -42 | | | -36 | | --- | | -36 | | | -16 | | --- | | -16 | | | -2 | | --- | | -8 | |
| 3 | Abscisic acid | 2.3 | [M-H]^-^ | 263.3 | 111 (9), 125 (8), 138 (10), 148 (4), 153 (100), 163 (11), 175 (2), 186 (4), 201 (25), 204 (43), 219 (60), 248 (2), 263 (44) | **263 → 153**  263 → 219 | | -18 | | --- | | -22 | | | -80 | | --- | | -80 | | | -21 | | --- | | -21 | | | -2 | | --- | | -2 | |
| 4 | Jasmonic acid | 2.8 | [M-H]^-^ | 209.0 | 59 (100), 97 (2), 109 (3), 165 (6), 209 (69) | **209 → 59**  209 → 41 | | -26 | | --- | | -54 | | | -60 | | --- | | -60 | | | -19 | | --- | | -19 | | | -6 | | --- | | -4 | |
| 5 | Jasmonic acid isoleucine conjugate | 3.2 | [M-H]^-^ | 322.4 | 129 (23), 130 (100), 172 (8), 322 (72) | **322 → 130**  322 → 172 | | -36 | | --- | | -22 | | | -54 | | --- | | -54 | | | -23 | | --- | | -23 | | | -4 | | --- | | -2 | |
| 6 | 12-Oxophytodienoic acid | 4.7 | [M-H]^-^ | 291.4 | 165 (20), 247 (17), 273 (7), 291 (100) | **291 → 165**  291 → 247 | | -26 | | --- | | -26 | | | -54 | | --- | | -48 | | | -22 | | --- | | -22 | | | -2 | | --- | | -3 | |
| A | ^2^H_6_-Salicylic acid | 1.7 | [M-H]^-^ | 142.9 | 69 (2), 98 (100), 142 (65) | **142** → 98 | | -20 | | --- | | | -36 | | --- | | | -16 | | --- | | | -2 | | --- | |
| B | ^2^H_6_-Abscisic acid | 2.3 | [M-H]^-^ | 269.9 | 114 (3), 130 (8), 143 (13), 159 (100), 169 (16), 181 (2), 192 (7), 207 (25), 210 (50), 225 (60), 269 (36) | **269 → 159** | | -18 | | --- | | | -80 | | --- | | | -21 | | --- | | | -2 | | --- | |
| C | ^2^H_6_-Jasmonic acid | 2.8 | [M-H]^-^ | 215.0 | 59 (40), 171 (10), 215 (100) | **215 → 59** | | -26 | | --- | | | -60 | | --- | | | -19 | | --- | | | -6 | | --- | |
| D | ^2^H_2_-Jasmonic acid-isoleucine | 3.2 | [M-H]^-^ | 324.4 | 130 (57), 174 (10), 324 (100) | **324 →** 130 | | -36 | | --- | | | -54 | | --- | | | -23 | | --- | | | -4 | | --- | |
| E | ^2^H_5_-12-Oxophytodienoic acid | 4.6 | [M-H]^-^ | 296.2 | 170 (12), 252 (29), 278 (5), 296 (100) | **296 →** 170 | | -26 | | --- | | | -54 | | --- | | | -22 | | --- | | | -2 | | --- | |

Analyses were performed on an ESI-QqLIT mass spectrometer operating in negative ion mode coupled on-line to a RP-UPLC system.

^a^ The analytes are listed in the order of their elution. ^b^ The MS/MS spectra were acquired at a collision potential of -20 V in negative mode.

^c^ Only signals with relative intensities of more than 2% of the base peak are shown. ^d^ Precursor ions are underlined. ^e^ The transitions used for quantification are in bold characters.

**Table A-2** Solid phase materials analyzed in a recovery screening experiment

| **Label** | **Commercial name** | **Producer** | **Chemistry** |
| --- | --- | --- | --- |
| I | Chromabond C18ec | Macherey-Nagel, Düren, Germany | silica-based RP |
| II | Strata C18-E | Phenomenex, Aschaffenburg, Germany | silica-based RP |
| III | Spec C18 AR | Agilent technologies, Böblingen, Germany | silica-based RP |
| IV | Cromabond HR-X | Macherey-Nagel, Düren, Germany | polymer RP |
| V | OASIS HLB | Waters, Eschborn, Germany | polymer RP |
| VI | BOND ELUT PLEXA | Agilent technologies, Böblingen, Germany | polymer polar-functionalized |
| VII | Strata X-AW30u | Phenomenex, Aschaffenburg, Germany | polymer WAX |
| VIII | OASIS WAX | Waters, Milford, Massachusetts, US | polymer WAX |
| IX | Cromabond HR-XAW | Macherey-Nagel, Düren, Germany | polymer WAX |
| X | OASIS MCX | Waters, Eschborn, Germany | polymer MCX |
| XI | OASIS WCX | Waters, Eschborn, Germany | polymer WCX |

RP Reversed Phase

WAX Weak Anion eXchange

WCX Weak Cation eXchange

MCX Mixed-mode Cation eXchange

**Table A-3** Analyte recoveries (%) in flow-through, wash and elution fractions observed after loading of standard phytohormone mixture.

| **Solid phase**  **material** | **Recovery (%) / Fraction** | | | | | | | | | | | | | | | | | |
| --- | --- | --- | --- | --- | --- | --- | --- | --- | --- | --- | --- | --- | --- | --- | --- | --- | --- | --- |
|  | **Flow-through** | | | **Wash** | | | **Eluate**  **acetonitrile** | | | **Eluate**  **isopropanol** | | | **Eluate**  **isopropanol/ 5% NH_3_** | | | **Eluate**  **isopropanol/ 5% HCOOH** | | |
|  | **SA** | **ABA** | **JA** | **SA** | **ABA** | **JA** | **SA** | **ABA** | **JA** | **SA** | **ABA** | **JA** | **SA** | **ABA** | **JA** | **SA** | **ABA** | **JA** |
| I | 0.04 ±0.01 | 1.92 ±2.15 | 7.06 ±8.55 | 31.40 ±1.59 | 0.90 ±0.27 | 6.38 ±2.55 | 41.90 ±6.92 | 64.26 ±3.76 | 121.46 ±13.87 | 1.12 ±0.07 | 0.02 ±0.03 | 0.04 ±0.03 | 0.37 ±0.11 | 0.01 ±0.03 | 0.00 ±0.00 | 0.88 ±0.20 | 0.02 ±0.03 | 0.02 ±0.01 |
| II | 5.92 ±2.90 | 5.20 ±2.64 | 9.54 ±4.65 | 16.01 ±5.77 | 0.00 ±0.00 | 0.00 ±0.01 | 64.64 ±10.50 | 58.58 ±12.51 | 103.36 ±9.30 | 4.15 ±1.22 | 1.55 ±0.73 | 2.48 ±1.30 | 1.16 ±0.38 | 0.02 ±0.03 | 0.01 ±0.01 | 1.30 ±1.09 | 0.01 ±0.02 | 0.02 ±0.01 |
| III | 0.05 ±0.05 | 0.00 ±0.00 | 0.00 ±0.00 | 31.33 ±1.61 | 21.43 ±3.14 | 27.69 ±0.57 | 14.26 ±1.72 | 37.65 ±8.67 | 49.75 ±6.48 | 1.68 ±0.22 | 0.93 ±0.30 | 0.93 ±0.30 | 0.61 ±0.49 | 0.00 ±0.00 | 0.01 ±0.01 | 1.04 ±0.61 | 0.00 ±0.00 | 0.02 ±0.00 |
| IV | 0.06 ±0.01 | 0.12 ±0.06 | 2.62 ±2.78 | 0.07 ±0.03 | 0.00 ±0.00 | 0.00 ±0.00 | 16.31 ±1.82 | 34.99 ±4.99 | 73.31 ±13.70 | 23.23 ±5.97 | 7.22 ±1.57 | 26.31 ±6.74 | 5.35 ±2.22 | 0.03 ±0.03 | 0.29 ±0.06 | 6.26 ±0.29 | 0.02 ±0.03 | 0.07 ±0.04 |
| V | 14.29 ±3.19 | 0.00 ±0.00 | 0.01 ±0.01 | 0.06 ±0.02 | 0.00 ±0.00 | 0.00 ±0.00 | 60.54 ±3.74 | 37.02 ±7.11 | 71.07 ±11.32 | 7.95 ±0.51 | 18.55 ±10.31 | 34.69 ±19.44 | 1.48 ±0.71 | 0.28 ±0.24 | 0.85 ±0.30 | 3.59 ±0.33 | 0.10 ±0.02 | 0.14 ±0.04 |
| VI | 5.26 ±0.71 | 0.14 ±0.06 | 0.89 ±0.30 | 0.63 ±0.20 | 0.00 ±0.00 | 0.00 ±0.00 | 54.99 ±2.89 | 54.33 ±2.26 | 124.38 ±6.36 | 15.02 ±4.12 | 2.67 ±0.65 | 4.57 ±1.54 | 1.05 ±0.22 | 0.26 ±0.31 | 0.79 ±1.16 | 2.04 ±1.35 | 0.10 ±0.08 | 0.06 ±0.03 |
| VII | 0.06 ±0.05 | 0.00 ±0.00 | 0.00 ±0.00 | 0.16 ±0.04 | 0.00 ±0.00 | 0.00 ±0.00 | 1.01 ±0.10 | 0.00 ±0.00 | 0.01 ±0.01 | 0.80 ±0.31 | 8.62 ±0.50 | 25.46 ±1.21 | 16.17 ±19.26 | 30.43 ±10.12 | 59.09 ±2.15 | 5.16 ±0.76 | 0.95 ±0.21 | 3.63 ±1.02 |
| VIII | 8.90 ±6.91 | 4.69 ±4.02 | 8.89 ±6.28) | 0.00 ±0.00 | 0.00 ±0.00 | 0.00 ±0.00 | 0.93 ±0.53 | 1.96 ±0.49 | 23.68 ±2.69 | 0.83 ±0.29 | 32.57 ±0.69 | 63.18 ±8.20 | 8.81 ±2.45 | 12.14 ±4.83 | 13.08 ±2.23 | 6.28 ±0.70 | 0.17 ±0.03 | 0.18 ±0.04 |
| IX | 6.56 ±3.48 | 5.21 ±3.17 | 7.97 ±5.11 | 0.00 ±0.00 | 0.00 ±0.00 | 0.00 ±0.00 | 0.60 ±0.26 | 0.01 ±0.02 | 0.03 ±0.03 | 0.77 ±0.42 | 0.02 ±0.03 | 0.01 ±0.01 | 4.84 ±0.81 | 26.50 ±1.28 | 54.80 ±6.56 | 12.02 ±2.68 | 3.18 ±1.13 | 3.55 ±0.90 |
| X | 0.08 ±0.05 | 0.00 ±0.00 | 0.00 ±0.00 | 0.81 ±0.63 | 0.00 ±0.00 | 0.00 ±0.00 | 76.27 ±5.18 | 65.25 ±4.00 | 115.62 ±9.67 | 8.01 ±1.65 | 1.14 ±0.20 | 1.86 ±0.51 | 0.44 ±0.38 | 0.03 ±0.02 | 0.09 ±0.03 | 2.51 ±0.30 | 0.00 ±0.00 | 0.04 ±0.02 |
| XI | 0.11 ±0.07 | 0.00 ±0.00 | 0.00 ±0.00 | 0.05 ±0.01 | 0.00 ±0.00 | 0.00 ±0.00 | 0.82 ±0.57 | 22.34 ±2.89 | 76.50 ±16.22 | 17.87 ±1.06 | 11.45 ±2.61 | 17.17 ±3.59 | 1.21 ±0.51 | 0.03 ±0.03 | 0.18 ±0.05 | 5.12 ±2.00 | 0.10 ±0.02 | 0.12 ±0.02 |

A standard mixture containing 19.8 mg/L of SA, ABA and JA (100 µL) was applied on the solid phase materials of different chemistry and specificity: Chromabond C18ec (I), Strata C18-E (II), Spec C18 AR (III), Chromabond HR-X (IV), OASIS HLB (V), Bond Elut PLEXA (VI), Strata X-AW30u (VII), OASIS WAX (VIII), Chromabond HR-XAW (IX), OASIS MCX (X), OASIS WCX (XI).

**Table A-4** Instrument sensitivity and linearity parameters obtained for phytohormone standards ^a^

| **Label** | **LOD_i_**  **(mol)** | **LOQ_i_**  **(mol)** | **LDR_i_** | **Slope** | **Intercept** | **R^2^** |
| --- | --- | --- | --- | --- | --- | --- |
| 1 | 2.5E-17 | 5.0E-15 | 2.0E+2 | 1.18E+3 | 1.44E+4 | 0.993 |
| 2 | 1.0E-16 | 1.0E-15 | 1.0E+4 | 2.54E+2 | 5.89E+1 | 0.999 |
| 3 | 2.5E-16 | 1.0E-15 | 1.0E+4 | 2.41E+2 | 8.64E+2 | 0.997 |
| 4 | 2.5E-17 | 2.5E-15 | 2.0E+4 | 1.50E+2 | -1.55E+1 | 0.999 |
| 5 | 1.0E-15 | 5.0E-15 | 2.0E+4 | 8.12E+1 | -8.30+2 | 0.995 |
| 6 | 2.5E-15 | 2.5E-12 | 4.0E+2 | 8.13E-1 | 2.14E+4 | 0.999 |

^a^ The standard analyte solutions were prepared by serial dilution of the stock mixture containing 1 mmol/L of each individual standard. The dilutions were performed with 20% aq. methanol in 2.0 – 2.5-fold increment to obtain 23 concentrations steps (0.01nmol/L – 500 µmol/L).

LOD_i_, instrument limit of detection; LOQ_i_, instrument limit of quantification; LDR_i_, instrument linear dynamic range

**Table A-5 Analyte recoveries observed with different drying – reconstitution strategies**

| **Drying**  **technique** | **Reconstitution**  **strategy** | **SA** | **ABA** | **JA** | **12-OH-JA** | **JA-Ile** | **OPDA** |
| --- | --- | --- | --- | --- | --- | --- | --- |
| Speed Vac**^a^** | H_2_O | 0.65 | 0.87 | 0.62 | 0.73 | 0.63 | 0.21 |
| Nitrogen stream**^b^** | H_2_O | 0.80 | 0.88 | 0.91 | 0.84 | 0.90 | 0.52 |
| Freeze-drying**^c^** | H_2_O | 0.74 | 0.70 | 0.76 | 0.69 | 0.70 | 0.57 |
| Speed Vac**^a^** | 50% aq. CH_3_CN | 0.59 | 0.46 | 0.60 | 0.65 | 0.76 | 0.55 |
| Nitrogen stream**^b^** | 50% aq. CH_3_CN | 0.70 | 0.76 | 0.95 | 0.73 | 0.81 | 0.77 |
| Freeze-drying**^c^** | 50% aq. CH_3_CN | 0.55 | 0.62 | 0.82 | 0.61 | 0.58 | 0.62 |
| Speed Vac**^a^** | CH_3_CN | 0.15 | 0.63 | 0.67 | 0.75 | 0.77 | 0.84 |
| Nitrogen stream**^b^** | CH_3_CN | 0.21 | 0.73 | 0.80 | 0.82 | 0.76 | 0.78 |
| Freeze-drying**^c^** | CH_3_CN | 0.30 | 0.69 | 0.81 | 0.77 | 0.75 | 0.80 |
| Speed Vac**^a^** | 50% aq. CH_3_OH | 0.70 | 0.76 | 0.31 | 0.94 | 0.86 | 0.27 |
| Nitrogen stream**^b^** | 50% aq. CH_3_OH | 0.80 | 0.83 | 0.49 | 1.13 | 0.91 | 0.70 |
| Freeze-drying**^c^** | 50% aq. CH_3_OH | 0.74 | 0.62 | 0.44 | 0.81 | 0.69 | 0.65 |
| Speed Vac**^a^** | CH_3_OH | 0.68 | 0.68 | 0.68 | 0.86 | 0.77 | 0.79 |
| Nitrogen stream**^b^** | CH_3_OH | 0.54 | 0.85 | 0.73 | 0.75 | 0.73 | 0.90 |
| Freeze-drying**^c^** | CH_3_OH | 0.62 | 0.82 | 0.82 | 0.96 | 0.72 | 0.89 |

^a^ Concentrator 5301 (Eppendorf, Hamburg, Germany)

^b^ Techne Sample Concentrator (**Bibby Scientific Limited**, Staffordshire, UK)

^c^ Christ LT-105 (Martin Christ Gefriertrocknungsanlagen GmbH, Osterode am Harz, Germany)

**Table A-6** Contents of phytohormones (pmol/g fresh weight, n = 5) in various plant species, developmental stages and organs determined with the novel LC-MS/MS-based method

| **Species** | **Plant organ** | **Individual phytohormones** | | |
| --- | --- | --- | --- | --- |
|  |  | **OPDA (pmol/g)** | **JA (pmol/g)** | **JA-Ile (pmol/g)** |
| *Oryza sativa*^a^ | root | 225 ± 92 | 51 ± 19 | 115 ± 15 |
| *Arabidopsis thaliana* (Col-O)^b^ | seedling | 2337 ± 1181 | 40 ± 19 | 122 ± 15 |
| *A. thaliana* (Col-O)^c^ | rosette leaf | 5380 ± 1814 | 117 ± 49 | 110 ± 28 |
| *Medicago truncatula* (A17)^d^ | leaf | 2982 ± 1208 | 21 ± 7 | 49 ± 12 |
| *M. truncatula* (A17)^d^ | root | 92 ± 33 | 19 ± 8 | 38 ± 9 |

^a^ For comparison with published data obtained with other methods see the reference [38] in the manuscript

^b^ For comparison with published data obtained with other methods see the reference [39] in the manuscript

^c^ For comparison with published data obtained with other methods see the reference [40] in the manuscript

^d^ For comparison with published data obtained with other methods see the reference [12] in the manuscript


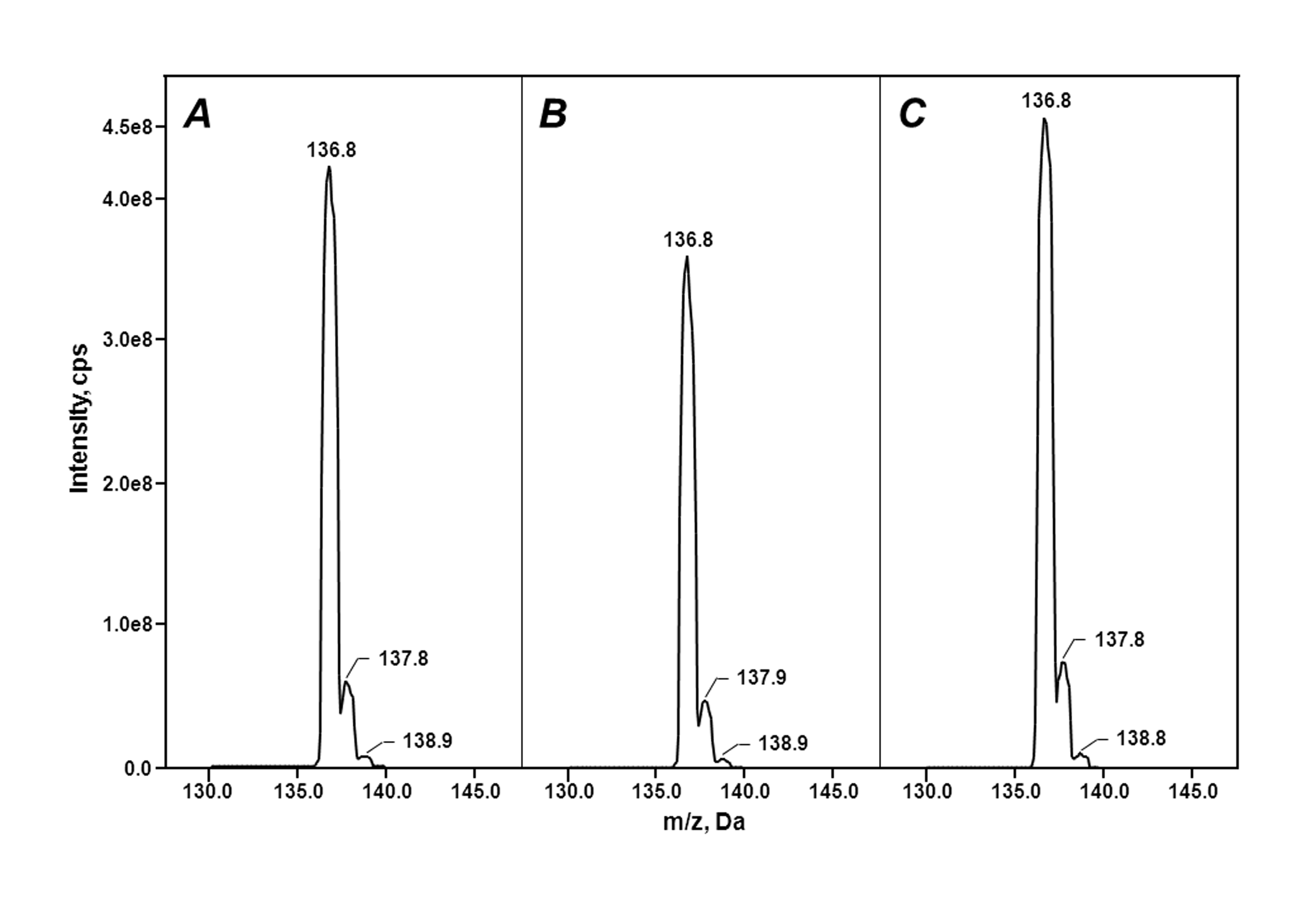


**Figure A-1** Intensities of salicylate [M-H]^-^ ion (*m/z* 136.8) observed in Q1 scans acquired with 1 µmol/L salicylic acid in 0.3 mmol/L formic acid, pH 2.7 (Panel A), 0.3 mmol/L ammonium acetate, pH 4.0 (Panel B) and 0.3 mmol/L ammonium formate, pH 3.5 (Panel C). The spectra were acquired with a QqLIT mass spectrometer operating in negative ion mode. Sample introduction was performed by syringe pump infusion at 15 µL/min.


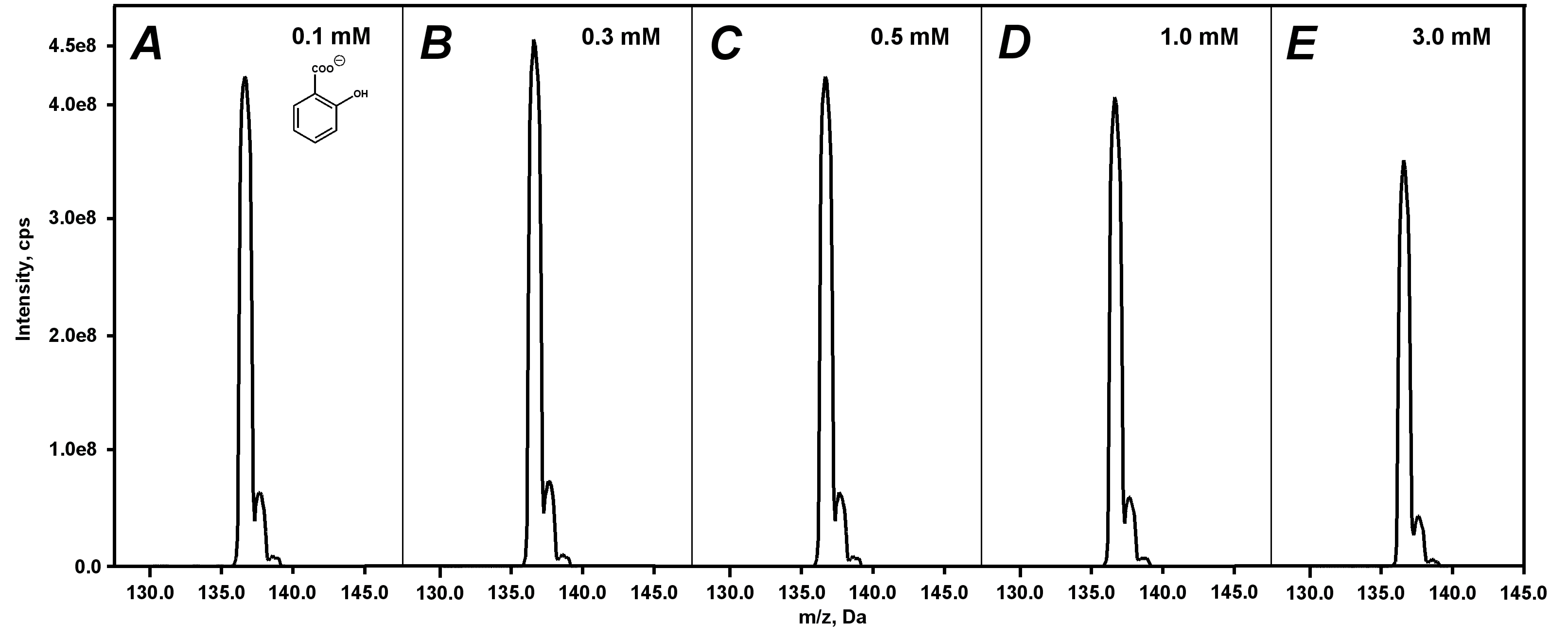


**Figure A-2** Intensities of salicylate [M-H]^-^ ion (*m/z* 136.8) observed in Q1 scans acquired with 1 µmol/L salicylic acid in 0.1 mmol/L (Panel A), 0.3 mmol/L (Panel B), 0.5 mmol/L (Panel C), 1.0 mmol/L (Panel D) and 3.0 mmol/L (Panel E) ammonium formate (pH 3.5). The spectra were acquired with a QqLIT mass spectrometer operating in negative ion mode. Sample introduction was performed by syringe pump infusion at 15 µL/min.

**
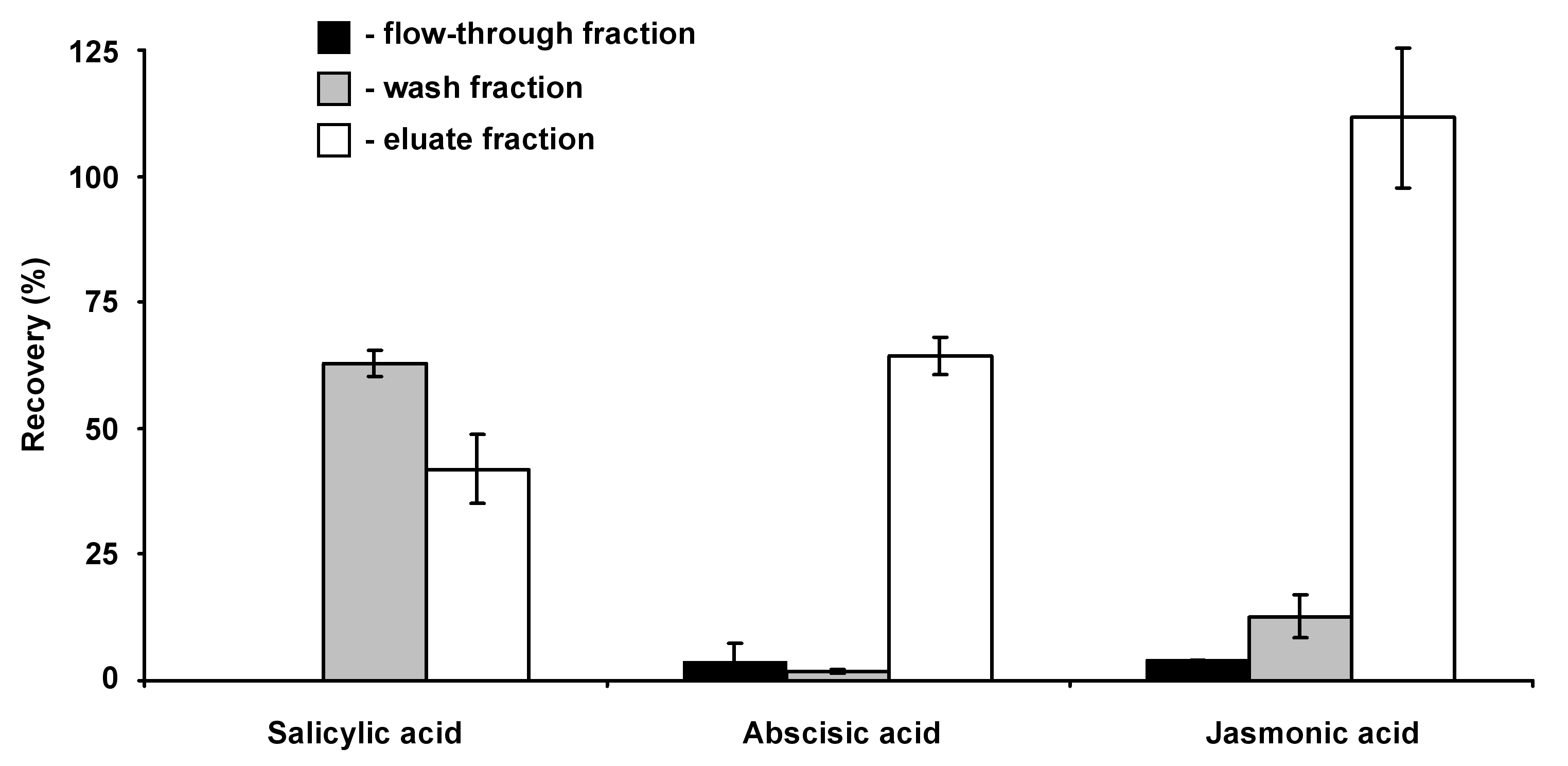
**

**Figure A-3** Recoveries of salicylic, abscisic and jasmonic acids from Chromabond C18ec SPE cartridges (50 mg material). The standard mixture (200 µg/mL in 10% aq. methanol) was applied on 50 mg cartridges and flow-through (10% aq. methanol), wash (water) and eluate (acetonitrile) fractions were collected and subjected to RP-UPLC-MS/MS. Recoveries were calculated from integrated MRM extracted ion chromatograms relative to untreated standard mixture.

**
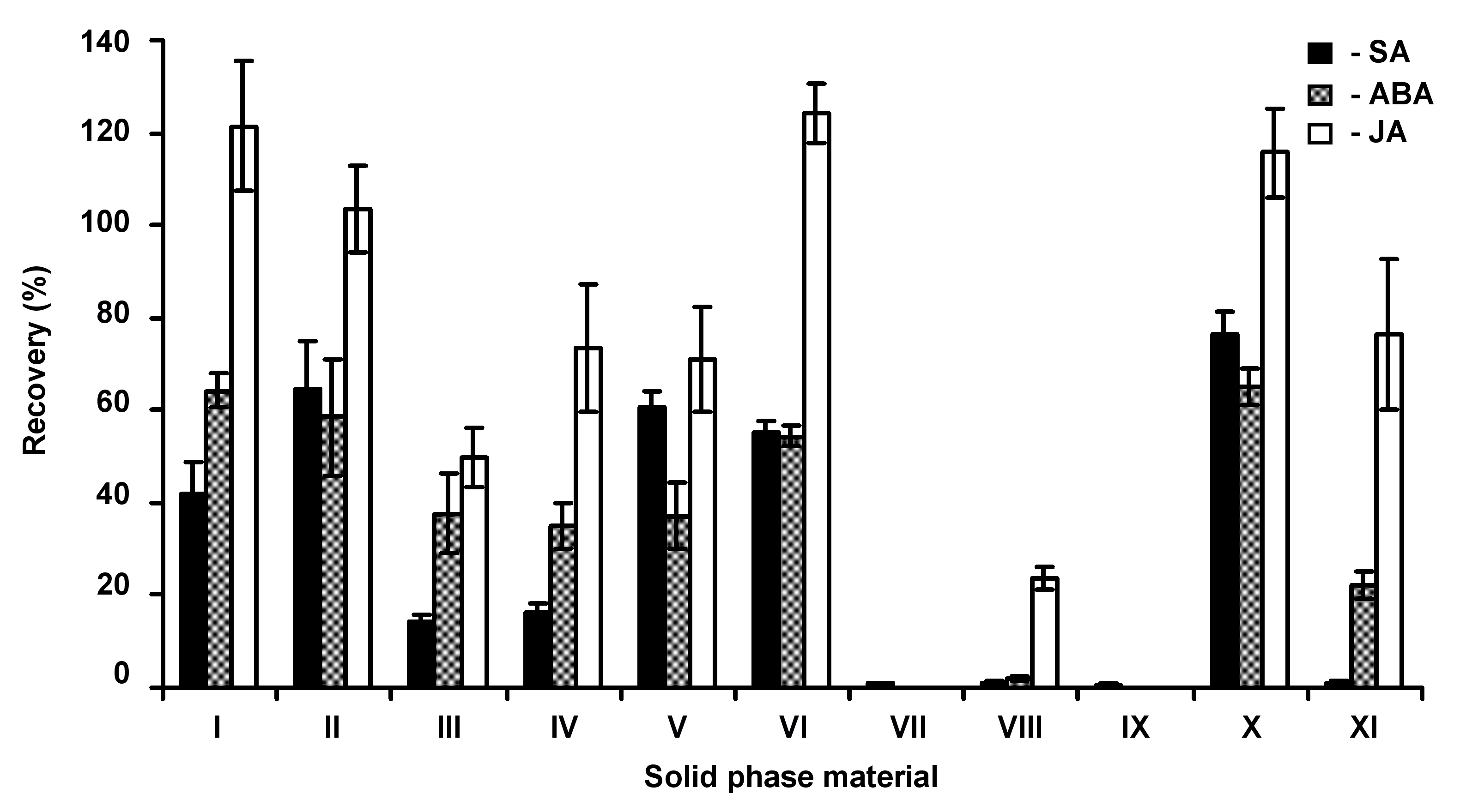
**

**Figure A-4** Phytohormone recoveries in acetonitrile elution fraction observed after loading of a standard mixture containing 19.8 mg/L of SA, ABA and JA (100 µL) on solid phase material of different specificity: Chromabond C18ec (I), Strata C18-E (II), Spec C18 AR (III), Chromabond HR-X (IV), OASIS HLB (V), Bond Elut PLEXA (VI), Strata X-AW30u (VII), OASIS WAX (VIII), Chromabond HR-XAW (IX), OASIS MCX (X), OASIS WCX (XI).
